# Mesenteric cysts and mesenteric venous thrombosis leading to intestinal necrosis in pregnancy managed with laparotomy: a case report and review of the literature

**DOI:** 10.1186/s13256-017-1320-5

**Published:** 2017-07-07

**Authors:** Aris Giannos, Sofoklis Stavrou, Nikolaos Goumalatsos, George Fragkoulidis, Eleni Chra, Dimitrios Argiropoulos, Dimitrios Loutradis, Peter Drakakis

**Affiliations:** 1 1st OB/GYN Department, School of Medicine, National and Kapodistrian University of Athens, Alexandra Hospital, Lourou and Vasilissis Sofias Ave, 11528 Athens, Greece; 2 2nd Department of Surgery, Aretaieio Hospital, National and Kapodistrian University of Athens, Athens, Greece; 3 Department of Pathology, Alexandra Hospital, Athens, Greece; 4 Department of Radiology, Alexandra Hospital, Athens, Greece

**Keywords:** Mesenteric cysts, Lymphatic cysts, Mesenteric vein thrombosis, Pregnancy, Acute abdominal pain, Intestinal ischemia, Laparotomy, Pulmonary thromboembolism

## Abstract

**Background:**

Mesenteric cyst is a rare clinical entity especially in pregnancy; therefore, few cases have been reported in the literature. The standard method of their treatment is surgical excision either with laparotomy or laparoscopy. In addition, mesenteric vein thrombosis is a rare and life-threatening condition in pregnancy and needs immediate treatment because it can lead to intestinal necrotic ischemia. This is the first report of the coexistence of mesenteric cysts and mesenteric vein thrombosis during gestation.

**Case presentation:**

A 27-year-old Greek woman, gravida 2 para 1, presented at 10 weeks’ gestation to the Emergency Unit of our hospital complaining of diffuse abdominal pain which deteriorated the last 3 days, which was localized in her right iliac fossa, along with vomiting. She had undergone open laparotomy and right salpingo-oophorectomy at the age of 23 due to an ovarian cyst. Besides this, her personal and family medical history was unremarkable. She had never received oral contraceptives or any hormone therapy. On arrival, a clinical examination revealed tenderness on palpation of her right iliac fossa, without rebound tenderness or muscle guarding. Within 10 hours of hospitalization, her symptoms deteriorated further with rebound tenderness during the examination, tachycardia, and a drop of 12 units in her hematocrit value. An emergency laparotomy was performed. Two mesenteric cysts and a 60 cm necrotic part of her intestine were revealed intraoperatively. In the postoperative period, she complained of acute abdominal pain, tachycardia, and dyspnea. Computed tomography imaging revealed mesenteric vein thrombosis and pulmonary thromboembolism. She was treated with low molecular weight heparin and she was discharged on the 11th postoperative day.

**Conclusions:**

To the best of our knowledge, this is the first report in the literature of a simultaneous mesenteric cyst and mesenteric vein thrombosis in pregnancy. It is known that pregnancy is a state of hypercoagulation and clinicians should bear in mind this rare clinical condition in their diagnostic algorithm for acute abdominal pain.

## Background

Mesenteric cysts and cystic mesenteric tumors constitute a very rare abdominal entity. The location of these cysts can be anywhere in the mesentery, from duodenum to rectum [1]. Since the first description by Benevieni in 1507, only a few cases have been described according to the literature. Al-Mulhim, in 2003, described the sixth case of mesenteric cyst during pregnancy which was managed laparoscopically for the first time [2]. Mesenteric cysts are often asymptomatic; however, they may present with acute abdominal pain in cases of infection or ruptured cysts. They constitute an umbrella definition, which includes lymphangiomas, benign and malignant cystic lymphangiomas, enteric cysts, dermoid cysts, and pseudocysts [3]. Imaging modalities such as ultrasonography, computed tomography (CT) scan, and magnetic resonance imaging (MRI) play a pivotal role in the diagnosis of mesenteric cysts. Surgical excision of the cyst is the treatment of choice either by laparotomy or laparoscopy. We report a very rare case of two mesenteric lymphatic cysts causing jejunal ischemia in a 27-year-old pregnant woman treated by laparotomy.

## Case presentation

A 27-year-old Greek woman, gravida 2, para 1, presented at 10 weeks’ gestation to our hospital complaining of diffuse abdominal pain for a week with deterioration the last 3 days and localization in her right iliac fossa, with simultaneous vomiting (April, 2016). Her medical history was unremarkable, although she had undergone laparotomy and right salpingo-oophorectomy at the age of 23 due to an ovarian cyst.

On arrival, a clinical examination revealed tenderness on palpation of her right iliac fossa, without rebound tenderness or muscle guarding. She had a temperature of 36.2 °C, her blood pressure was 154/97 mmHg, and her pulse rate was 97 beats per minute. Her body mass index (BMI) was 37.1 kg/m^2^. Her respiratory rate was normal. A cardiac examination showed S1, S2, mild tachycardia, and no murmur. A thorough neurological examination was performed; she was alert, attentive, and oriented. Her speech was clear and fluent with good repetition, comprehension, and naming. In addition, muscle bulk and tone were normal. Leukocytosis (white blood cell count 27,400/μl) and increased serum C-reactive protein (185 mg/dl) were the only pathologic findings. Obstetrical and gynecological causes were the first to consider; therefore, we tried to make an evaluation of the patient by transvaginal ultrasonography excluding causes of acute abdominal pain such as placenta disruption, abortion, ectopic pregnancy, or huge myomas which may have led to this deterioration. An intrauterine pregnancy with a live fetus and normal bilateral adnexa were revealed. A vaginal examination and surgical physical evaluation were normal.

**Fig. 1:**
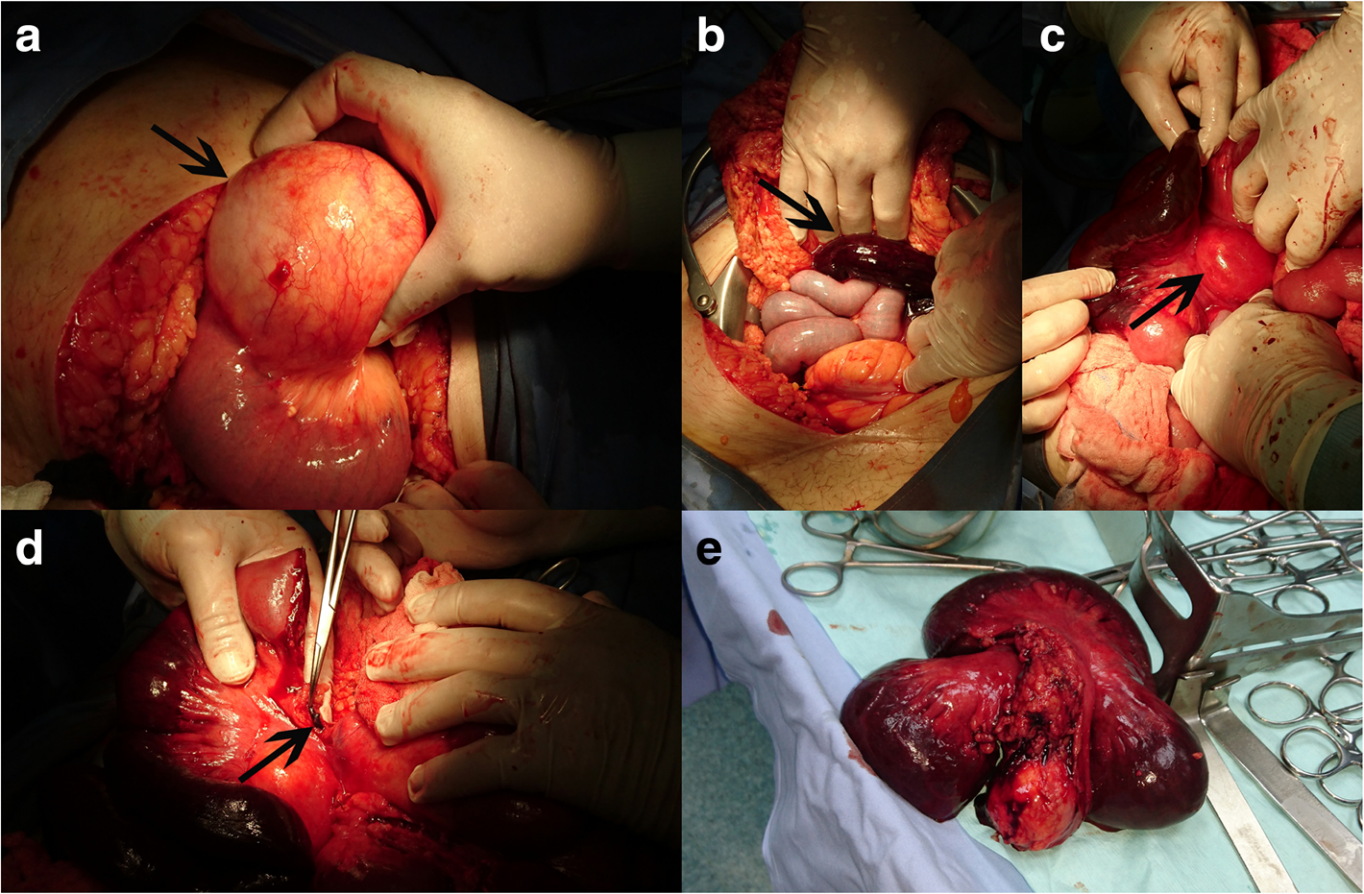
Intraoperative images. **a** Big lymphatic mesenteric cyst, as indicated by *black arrow*, at start of surgery; **b** part of necrotic intestine and part of normal intestine (as indicated by *black arrow*); **c** part of ischemic intestine and simultaneously the presence of the smaller lymphatic mesenteric cyst (as indicated by *black arrow*); **d** thrombosed mesenteric vessel after the resection of necrotic intestinal part (as indicated by *black arrow*); **e** the necrotic intestinal part of 60 cm

Within 10 hours of hospitalization her symptoms deteriorated further with rebound tenderness, tachycardia (130 beats per minute), and a drop of 12 units in her hematocrit level. A transvaginal ultrasonography showed accumulation of free fluid in the pouch of Douglas, with normal left adnexa; a transabdominal ultrasound of her upper and lower abdomen by radiologists revealed a cyst approximately 9 cm diameter which was of unknown origin. Due to her increased BMI, her history of a previous laparotomy, and the lack of available qualified specialists for laparoscopy at that time, an immediate emergency laparotomy was decided (23 April 2016; Fig. 1); under general anesthesia and a lower midline incision, two cysts of 9 cm and 4 cm diameter were found in the mesentery of the jejunum combined with thrombosis in her jejunal arteries (the presence of thrombus was observed macroscopically by surgeons behind the smaller cyst) and consequent jejunal ischemia (60 cm necrotic bowel), while free hemorrhagic fluid was found in her abdominal cavity. The excision of both cysts was performed; the ischemic small intestine was resected (60 cm of ischemic intestine) and a primary anastomosis was performed. Finally, an appendectomy was done due to the hemorrhagic and necrotic “appearance” of her appendix.

Postoperatively, she was kept nil by mouth; she had a Levin catheter and urine catheter for daily output measurement. The first postoperative day she had a high fever (up to 38.5 °C) and was treated with piperacillin and tazobactam (Tazocin; 4.5 gr × 3). She was mobilized very early and wore antithrombotic socks continuously; she was administered low molecular weight heparin (LMWH) with tinzaparin by injection (Innohep) 5000 IU subcutaneous injection once per day. Blood cultures revealed *Staphylococcus* species sensitive to the antibiotics she was taking. Her hemoglobin was 9.5 g/dl. On the third postoperative day, 2 units of red blood cells were transfused because of her low hematocrit of 21, 9% and hemoglobin of 7 g/dl. She complained of abdominal pain, so an abdominal ultrasonography was performed; an intrauterine pregnancy with a live embryo and an enlarged spleen with maximum diameter of 19 cm were revealed. The fourth postoperative day she had vaginal bleeding leading to incomplete abortion of the fetus. So, she underwent obstetric curettage successfully.

**Fig. 2:**
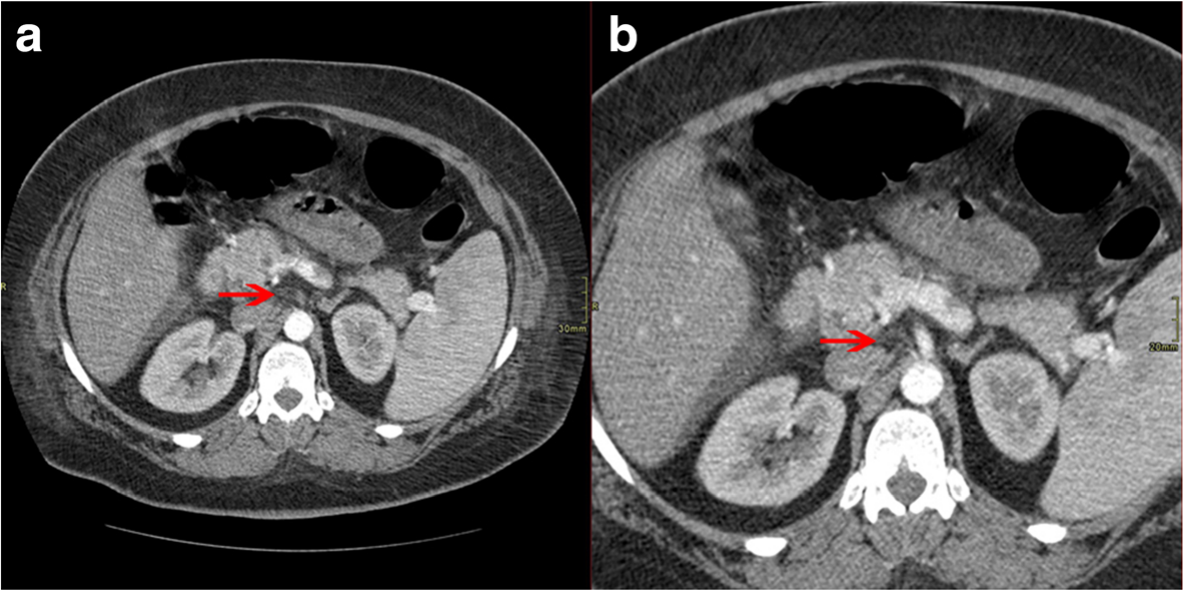
Abdominopelvic computed tomography scan. **a**, **b** Filling defect – thrombus is revealed in junction of liver–spleen axis and upper mesenteric vein (*red arrows*)

A few hours after the procedure, she had tachycardia (130 beats per minute), respiratory rate of 25 breaths per minute, dyspnea without chest pain, and a blood pressure of 121/76 mmHg. An electrocardiogram (ECG) revealed sinus tachycardia; her fibrinogen was 3.6 g/dl, activated partial thromboplastin time (APTT) 35 seconds, international normalized ratio (INR) 1.16, prothrombin time (PT) 13.4 seconds, pH 7.48, partial pressure of carbon dioxide (pCO_2_) 29 mmHg, partial pressure of oxygen (pO_2_) 63 mmHg, bicarbonate anion (HCO_3_^-^) 21.6 mmol/L, oxygen saturation (SO_2_) 94%, and her serum level of d-dimers was 28,132 mg/l. Based on the fact that she was pregnant, that she had recent surgery, the presentation of thrombosis during surgery, her obesity, and her clinical symptoms, an emergency thorax CT scan for the possibility of pulmonary embolism (CT pulmonary angiogram) was ordered, while the dosage of LMWH (tinzaparin by injection) was increased to 7000 IU subcutaneous injection once per day. The thorax CT scan showed embolism to branches of her pulmonary artery, small pleural effusion with atelectasis in the dorsal part of her lungs, and small pericardial effusion. A supplementary abdominopelvic CT scan with an injection of iodine-rich contrast material administered intravenously revealed a small filling defect – thrombus – in the celiac axis of her abdominal aorta, mesenteric vein thrombosis (MVT) was revealed as filling defect in her mesenteric vein, as well as filling defect in her common iliac vein and in the initial portion of her left external iliac vein (Fig. 2). So the decision for an increased suitable anticoagulant dosage was taken; the new therapy was enoxaparin by injection (LMWH) 8000 IU subcutaneous injection twice daily. The triplex of veins and arteries of her legs was normal, as well as the color-flow Doppler echocardiography. Her family history for thrombosis was unremarkable. She had never used any oral contraceptives or similar hormonal therapy in the past.

**Fig. 3:**
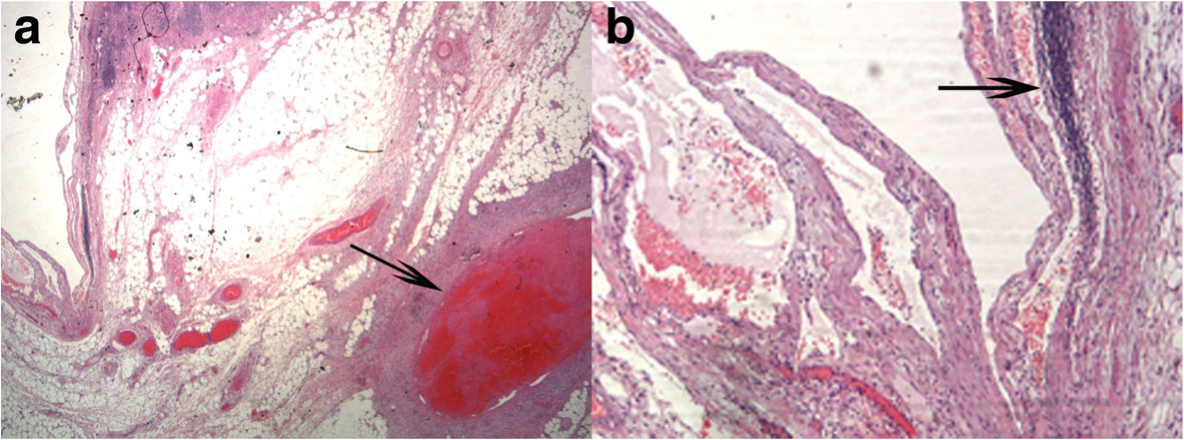
Histopathological images after hematoxylin and eosin stain. **a** Thrombosed vascular vein branch (*black arrow*) near the lymphatic cyst; **b** Presence of lymphatic tissue (*black arrow*)

During hospitalization in our intensive care unit, the results of a thrombophilia investigation were normal; more specifically, she was negative for prothrombin F20210A mutation and factor V (FV) Leiden mutation, heterozygous for methylene tetrahydrofolate eductase (MTHFR) C677T mutation, and heterozygous for MTHFR C1298T mutation. A paroxysmal nocturnal hemoglobinuria (PNH) test was negative. The results for lupus anticoagulant, anti-cardiolipin antibodies (ACA), and anti-beta-2-glycoprotein I (Aβ2GpI) antibodies (IgG and IgM) were all in normal ranges. Protein S and protein C deficiency had normal values. She was discharged on the 11th postoperative day with normal blood and urine tests. A histological diagnosis confirmed two lymphatic cysts, ischemic intestinal necrosis, hemorrhagic mesenteric fat, and a hemorrhagic and swollen appendix; a thrombosed vascular venous branch was observed in the mesenteric adipose tissue proximate to the smaller cyst (Fig. 3).

She was discharged with instructions to take one tablet of rivaroxaban 20 mg (Xarelto) administered orally once per day for 6 months. She continues to receive this anticoagulant therapy, although she does not complain of any suspicious symptoms. She is in continual communication with surgeons, gynecologists, and hematologists of our department and her next appointment will be in the next few days at the time of this writing.

## Discussion

Cysts of the mesentery were first described by Benevieni, a Florentine anatomist, in 1507 during an autopsy on an 8-year-old boy [4, 5]. The incidence of mesenteric cysts is 1/102,500 to 1/250,000 hospital admissions; it is identified in up to 1 in 20,000 acute pediatric admissions. Lambregts *et al*., in a recent paper (2014), described the seventh case of mesenteric cysts during pregnancy [6]; it was the second case, after that of Al-Mulhim [2], managed with laparoscopy [2, 6]. Almost all the cases were managed with laparotomy except the two cases in which laparoscopy was performed.

Several causes have been described for these cysts: some believe that their origin is from continual growth of congenitally malformed or malpositioned lymphatic tissue, while others propose theories such as lymph node degeneration or trauma theory in which mesenteric cysts develop secondarily. Cysts of the mesentery can be located from the duodenum to the rectum anywhere in the mesentery [7].

The cysts can be asymptomatic and on average 50% are detected incidentally [2] through abdominal ultrasonography, CT, or magnetic resonance imaging (MRI) [6]. Ultrasonography is a pivotal tool in the first-line assessment of mesenteric lesions and supplementation with a CT scan may help in the planning of the surgical approach [8]. The most common symptoms related with mesenteric cysts are abdominal pain and distension, according to the size and the location of the cysts, as well as the consequential abdominal organ compression (hydronephrosis, lower extremity lymphedema, intestinal obstruction). Acute abdominal pain can be presented in a few cases [2].

The classification proposed by de Perrot *et al*. in 2000 divides the mesenteric cysts in six groups, namely lymphatic cysts and lymphangiomas, cysts of mesothelial origin, cysts of enteric origin, cysts of urogenital origin, mature cystic teratomas (dermoid cysts), and non-pancreatic pseudocysts [5]. Their fluid can present as serous, chylous, sanguineous, or chylolymphatic; they can present as single cysts, septated cysts, or multilocular cysts radiographically [9]. Malignancy has been reported with an incidence of less than 3% [7].

Complete surgical excision is the treatment of choice for mesenteric cysts, either by laparoscopy or laparotomy [6].

Despite the fact that surgical operations should be avoided during pregnancy, non-obstetric laparotomy is performed in 1.6 to 2.2% of pregnancies. Pregnancy used to be a contraindication for laparoscopy but recent data show that laparoscopic procedures can be performed safely during pregnancy [2].

Pregnancy as a hypercoagulable state “is a normal situation associated with increased risk of venous thromboembolism.” Pregnant women have a fourfold to fivefold increased risk compared with non-pregnant women [10]. Factors VII, VIIIC, and fibrinogen are increased, while the activity of the fibrinolytic mechanism is reduced [11]. In addition, some factors such as protein S decrease to 40 to 60% of normal level in order to manage effective bleeding control during delivery [12].

Mesenteric venous thrombosis is very difficult to diagnose due to its nonspecific clinical symptoms, the most frequent complaint in cases of acute MVT is abdominal pain [10]; apart from that, it remains a rare localization of thrombosis. Similarly, in pregnancy, the diagnosis of MVT is difficult because some symptoms can wrongfully present as normal changes of pregnancy [13].

Mesenteric venous thrombosis is a rare cause of acute mesenteric ischemia, representing only 5 to 15% of patients [14]. MVT has been estimated to account for 0.002 to 0.06% of all in-patients admissions, 0.01% of all emergency surgical admissions, and less than 1 in 1000 laparotomies for “acute abdominal pain” [15]. In general, the most dangerous complication of MVT is intestinal ischemia, with mortality of 20 to 50% [13].

Intestinal ischemia after MVT is an uncommon entity during pregnancy. Lin *et al*. in a recent case report, after reviewing the literature, stated that only 16 cases of small bowel ischemia during pregnancy have been reported and only nine cases (56.2%) were associated with MVT [16].

Recently (2016), García-Botella *et al*. described the tenth case report in which, despite the existence of MVT and bowel resection laparoscopically, fetal viability was preserved, in this case for a pregnant woman in her 7th week of pregnancy [13].

Plenty of risk factors have been described for MVT including portal hypertension, postoperative complications, neoplasia, trauma, inflammatory bowel disease, malignancy, pregnancy, hemoglobinopathies, protein S deficiency, protein C deficiency, oral contraceptive agents, and the use of estrogen-containing compounds [16]. MVT due to FV Leiden mutation has been reported in recent studies, while the first case in pregnancy was demonstrated by Sönmezer *et al*. (2004) [17]. At least one of the above factors presents in most cases with MVT, while idiopathic MVT ranges from 21 to 49% among the different series [15].

The early diagnosis of MVT, which is a potentially life-threatening condition, is very important to prevent high mortality and morbidity [17]. Non-characteristic signs and symptoms in pregnancy are the common problems leading to late diagnosis and treatment of acute abdominal pain. Only the one-third of patients with acute mesenteric ischemia were correctly diagnosed before surgical procedure or death. Pregnancy and its complications, such as abortion and preterm labor, may mimic clinical signs of abdominal conditions which require surgical treatment [18].

Our case report is the 11th case in the literature of MVT during pregnancy which led to mesenteric ischemia, whereas it is the eighth case in the literature of a mesenteric cyst during pregnancy. According to the literature, this is the first case of a mesenteric cyst and simultaneous MVT in a pregnant woman.

In our case report, we cannot be sure of the exact cause of thrombosis and intestinal necrosis, but we guess that thrombosis is due either to the strangulation of the vessels from the lymphatic mesenteric cyst or it is idiopathic, without forgetting that pregnancy plays a pivotal role leading to MVT as a “hypercoagulable” situation.

## Conclusions

This is the first case in the literature of a simultaneous mesenteric cyst and MVT in a pregnant woman who initially presented with acute onset abdominal pain. Despite the rarity of this condition in the gestational period, clinicians should take into account that pregnancy is a state of hypercoagulation and the existence of a mesenteric cyst and MVT, a potentially life-threatening condition, should be ruled out in the diagnostic process even in women with no known thrombophilia.

## Abbreviations

Aβ2GpI: Anti-beta-2-glycoprotein I
ACA: Anti-cardiolipin antibodies
APTT: Activated partial thromboplastin time
BMI: Body mass index
CT: Computed tomography
ECG: Electrocardiogram
FV: Factor V
HCO_3_^-^: Bicarbonate anion
INR: International normalized ratio
LMWH: Low molecular weight heparin
MRI: Magnetic resonance imaging
MVT: Mesenteric vein thrombosis
PCO_2_: Partial pressure of carbon dioxide
PNH: Paroxysmal nocturnal hemoglobinuria
PO_2_: Partial pressure of oxygen
PT: Prothrombin time
SO_2_: Oxygen saturation
